# A suspicious role of interferon in the pathogenesis of SARS-CoV-2 by enhancing expression of ACE2

**DOI:** 10.1038/s41392-020-0185-z

**Published:** 2020-05-21

**Authors:** Shan Su, Shibo Jiang

**Affiliations:** Key Laboratory of Medical Molecular Virology (MOE/MOH/CAM), School of Basic Medical Sciences, Fudan University, 130 Dong An Rd., Xuhui District, Shanghai, 200032 China

**Keywords:** Infection, Molecular medicine

Based on a number of published and unpublished single-cell RNA-sequencing (scRNA-seq) datasets, a recent study identified putative specific cell subtypes targeted by SARS-CoV-2 (severe acute respiratory syndrome coronavirus 2) and a possible role of interferon (IFN) in its pathogenesis. The rapid spread of SARS-CoV-2, the causative pathogen of COVID-19, has had global public health and economic consequences. The molecular mechanism(s) underlying the high transmissibility of SARS-CoV-2 remain(s) elusive. Therefore, we must follow a pragmatic and stepwise path to determine the specific target cell subsets and factors that regulate SARS-CoV-2 infection to elucidate true targets for treatment. In a recent issue of *Cell*, Carly and co-workers^1^ identified the cell subsets targeted by SARS-CoV-2 in certain tissues. In addition, they found that IFN-α enhanced the expression of angiotensin-converting enzyme 2 (ACE2), the receptor for SARS-CoV-2, in primary human upper airway basal cells. However, we know that IFN is cell-type- and host species-specific, indicating the need for more preclinical and clinical data to clarify the role of IFN in the pathogenesis of COVID-19.

SARS-CoV-2 spike (S) protein-mediated viral entry requires binding to ACE2 and cleavage by host proteases, principally TMPRSS2 (transmembrane serine protease 2).^2^ Therefore, ACE2 and TMPRSS2 dual-positive cells are likely targets for SARS-CoV-2. By comparing the RNA profile of different cell subsets from diverse tissues of non-human primate (NHP), human, and mouse, Carly and co-workers^1^ found that ACE2 and TMPRSS2 are both enriched within epithelial cells, especially type II pneumocytes in the lung, absorptive enterocytes in the intestine, as well as goblet secretory cells in the nasal and sinus tissue. The detection of possible target cells for SARS-CoV-2 in the lung, nasal and sinus tissues, and the intestine is consistent with the clinical observation that SARS-CoV-2 RNA has been detected in sputum, nasopharyngeal swabs, and stool samples. Validation of cell types infected by SARS-CoV-2 would provide a theoretical basis for selecting a cell model to evaluate newly developed COVID-19 drugs and vaccines.

**Fig. 1:**
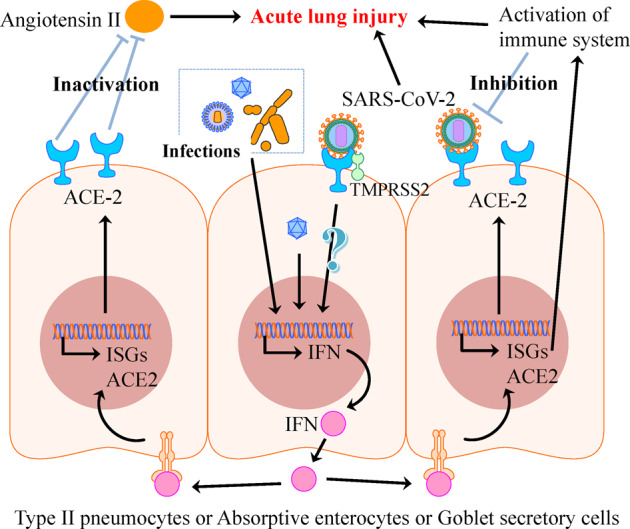
The multifunctional role of interferon (IFN) in the pathogenesis of SARS-CoV-2. Pathogenic infections induced expression of IFN, which then activated canonical IFN-induced genes (ISGs) and AEC2 expression. On the one hand, upregulation of ACE2 might result in more SARS-CoV-2 infection. On the other hand, ACE2 help to protect host from acute lung injury by inactivating angiotensin II. Other ISGs protein could further activate broad immune responses, which could not only inhibit SARS-CoV-2 infection, but also contribute to the acute lung injury by inducing inflammatory response

By comparing the differentially expressed genes between ACE2^+^ and ACE2^−^ cells, the investigators identified an association between ACE2 expression and canonical IFN-stimulated genes (ISGs), or components of the IFN signaling pathway (Fig. 1). ACE2^+^ cells are more prevalent in lung and ileum samples from chronically infected human or NHP in which IFN is typically upregulated to control infection. Therefore, the authors then investigated whether IFNs might also regulate ACE2 expression levels in specific target cell subsets. They found that type I and type II IFNs induced ACE2 expression in several published datasets of nasal epithelial cells, primary bronchial cells, and keratinocytes. The in vitro experiments further confirmed that ACE2 RNA and protein expression level were both elevated by IFN in primary human upper airway basal cells. According to the authors, “this discovery that ACE2 is an ISG in human epithelial cells, along with SARS-CoV-2 utilizing host ACE2 to gain entry to cells, suggests that SARS-CoV and SARS-CoV-2 may exploit the ACE2-mediated tissue-protective response to provide further cellular targets for entry.”

This conclusion has elicited intense discussion. A comparison between SARS-CoV-2 and HIV has arisen because both exploit the human immune system to enhance infection. On the one hand, ACE2 can act as a tissue-protective component during severe acute lung injury induced by other respiratory viruses, such as influenza and HCoV-OC43.^3^ Therefore, induction of ACE2 expression would not be a trait exclusive to SARS-CoV-2 and it is still elusive whether increasing expression of ACE2 is more protective or harmful in vivo. On the other hand, Kwok-Yung and co-workers^4^ reported that “SARS-CoV-2 did not significantly induce types I, II, or III interferons in the infected human lung tissues,”^4^ indicating that ACE2 might not be upregulated by SARS-CoV-2 since IFN is rarely induced. Thus, at this point, whether IFN plays an important role in the pathogenesis of SARS-CoV-2 needs more clinical and experimental investigation.

IFN is a widely used agent for treating viral diseases, such as hepatitis C virus and hepatitis B virus infection. It has been reported that pretreatment with pegylated IFN-α significantly reduced virus titer in the lungs of SARS-CoV-infected macaques.^5^ These findings suggest that IFN has been considered as a promising drug candidate for SARS-CoV-2. The study of Carly and co-workers^1^ has raised concerns about the safety of using IFN in the clinical treatment of SARS-CoV-2, but no clear conclusion was drawn. First, this study mainly focused on type I IFNs (IFNα, IFNβ) and type II IFNs (IFNγ), whereas type III IFNs (IFNλ) were not investigated. Second, as the author stated, the scRNA-seq datasets used in this study were not specifically designed to understand how IFN functions in the pathogenesis of SARS-CoV-2. Since the induction of ACE2 expression by IFN is cell-type-specific, it still cannot be concluded that cell subsets in which IFN upregulates ACE2 are the same as those targeted by SARS-CoV-2. Finally, as the author stated in a news report, “It’s hard to make any broad conclusions about the role of interferon against this virus. It’s a balance between host restriction, tissue tolerance, and viral enhancement mechanisms of IFN.”

Even though we cannot conclude the effect of IFN on the pathogenesis and treatment of SARS-CoV-2 now, this study has still provided some valuable information. For example, it has proposed that ACE2 is an ISG in primary human target cells, while previous studies were based on cell lines and did not find that ACE2 was an ISG. Second, ACE2 expression was not driven by IFN in a mouse model, highlighting the importance of careful selection of in vivo models for understanding the pathogenesis of SARS-CoV-2. Last, the possible dual roles of IFN in SARS-CoV-2 infection require close monitoring of patients’ condition in the clinical use of IFN as a treatment.
